# Just as I Expected: Experimentally Testing the Influence of Expectancies on Perceptions of Interrogations

**DOI:** 10.3390/bs16081401

**Published:** 2026-08-15

**Authors:** Christopher J. Normile, Kyle C. Scherr

**Affiliations:** 1 Department of Psychology, Allegheny College, 520 N. Main Street, Box 39, Meadville, PA 16335, USA; 2 Department of Psychology, Central Michigan University, Mt. Pleasant, MI 48859, USA; scher1kc@cmich.edu

**Keywords:** jury decision-making, exonerations, fundamental attribution error, expectations

## Abstract

Expecting that a future consequence will occur has a meaningful and powerful influence on people’s behavior, regardless of whether the expected event actually occurs. However, most people do not appreciate the substantial impact that expectations have on human behavior. In this series of studies, we investigated people’s ability to grasp the power of expectations on suspects’ confession decisions. In Experiment 1 (*N* = 253), mock-jurors failed to appreciate the power of expectation and believed that a defendant who confessed after being induced with an expectation regarding the length of an interrogation was more likely to be guilty than a defendant who actually experienced a lengthy interrogation. In Experiment 2 (*N* = 261), online participants were less likely to support reintegration services for an exoneree who falsely confessed after being induced with expectations during his interrogation compared to an exoneree who actually experienced a lengthy interrogation. Collectively, the results of Experiments 1 and 2 further our understanding of people’s difficulty in appreciating situational influences and, in particular, suspects’ decision-making and the subsequent effects of false confession evidence.

## 1. Introduction

False confessions are associated with about 25% of the approximately 642 exoneration cases involving DNA (National Registry of Exonerations, 2026). Forty years of research have identified the influence of numerous dispositional factors (including age and intelligence; Redlich & Goodman, 2003; Gudjonsson, 1991), situational factors (e.g., police tactics and time of day; Russano et al., 2005; Scherr et al., 2014), and cognitive processes (e.g., temporal discounting; Madon et al., 2012) on people’s willingness to confess to wrongdoing (for a review, see Kassin et al., 2025). Unfortunately, this body of research has also established that those false confessions have a ripple effect in that they continue to have consequences for the innocent confessor long after the interrogation concludes (Scherr et al., 2020). For instance, false confessions all but guarantee a conviction at trial (e.g., 81% of people who falsely confess are convicted during criminal trials; Drizin & Leo, 2004; Kassin et al., 2014; Wallace & Kassin, 2012) and are associated with significant delays to release and official exoneration (Scherr & Normile, 2022).

Although decades of research have established that false confessions occur, and a body of psychological science establishes how they occur (e.g., Kassin et al., 2010; Kassin et al., 2025), the aftereffects that persist after the innocent individual leaves the interrogation room are less well understood (for some examples see Clow & Leach, 2015; Luke & Alceste, 2020; Scherr & Normile, 2022). For instance, even though suspects who falsely confess during obviously high pressure and coercive interrogations are often, nonetheless, perceived as guilty (e.g., Kassin & Sukel, 1997; Wallace & Kassin, 2012), it remains unknown whether unique interrogation factors result in varying degrees of guilt judgments. Similarly, despite growing evidence highlighting that those exonerated of a wrongful conviction based on a false confession are more stigmatized than other exonerees (Clow & Leach, 2015; Kukucka & Evelo, 2019; Scherr et al., 2018), it remains unknown whether different interrogation factors leading to the false confession can result in meaningfully different degrees of stigma. Here, we tested the general idea that innocent individuals who confessed because they expected a long interrogation would be perceived and judged more negatively than individuals who confessed because of an objectively long interrogation.

### 1.1. The Power of Expectations

Expectancies refer to individuals’ beliefs that specific behaviors will lead to particular outcomes (Tolman, 1955). Born out across decades of research, expectancies meaningfully influence a host of practical outcomes (Beal & Crockett, 2010; Madon et al., 2009; Najdowski et al., 2015), including suspects’ confession decision-making (Madon et al., 2012). Indeed, interrogators commonly attempt to induce an expectancy to suspects that they will remain in the interrogation for an extended period of time—perhaps even indefinitely—if they continue to refuse confessing (Leo, 2008; Leo et al., 2009). As a consequence of the expectancy, suspects may begin to believe that if they comply with the interrogators’ requests, they will finally be able to escape the interrogation (Yang et al., 2016).

In one study, participants were interviewed about their involvement in former criminal and unethical behaviors in which denials led to an immediate proximal consequence (i.e., answering mundane follow-up questions) while admissions would lead to a more severe far-off distal consequence (i.e., discussing criminal activity with a police officer; Madon et al., 2012). Results indicated that the longer the interview lasted, the more likely the participants were to discount the more serious distal consequence in order to avoid the immediate consequence. This pattern is consistent with research on temporal discounting, which demonstrates that individuals systematically devalue distal outcomes relative to immediate consequences, particularly under conditions of uncertainty and psychological strain (Brogan et al., 2025; Critchfield & Kollins, 2001; White-Cascarilla et al., 2025). A follow-up study found that merely inducing an expectation of length was powerful enough to increase participants’ willingness to admit wrongdoing (Madon et al., 2013). That is, when participants were simply induced with the expectation that the questioning was going to last for a substantial amount of time, they engaged in similar myopic decision-making as participants actually questioned for a longer period of time.

Together, these findings suggest that expectancies can influence confession decision-making not only by shaping beliefs about likely outcomes, but also by altering the subjective value of those outcomes over time. This process in turn increases the appeal of immediate relief relative to more consequential, delayed costs. From this perspective, confession decisions may reflect boundedly rational responses (see Simon, 1955) to a constrained and psychologically taxing decision environment rather than fully informed, deliberate choices. However, although research has shown that expectancies can influence confession decision-making, it remains unknown whether triers-of-fact and laypeople can appreciate the influence of expectancies on suspects’ decision-making.

### 1.2. False Confessions and Jury Decision-Making

Confessions, even those that are later deemed false, are considered one of the most, and oftentimes the most, powerful pieces of incriminating evidence in the courtroom (Kassin et al., 2014; Kassin & Neumann, 1997; Wallace & Kassin, 2012). False confessions can even influence suspects’ fate before a trial by corrupting other pieces of evidence such as eyewitness identifications (Hasel & Kassin, 2009), alibi support (Marion et al., 2016), latent print analyses (Dror & Charlton, 2006), and DNA analyses (Dror & Hampikian, 2011). The contaminated evidence can be so compelling that jurors (Kassin & Sukel, 1997; Kassin & Wrightsman, 1980; Neuschatz et al., 2008) and experienced judges (Wallace & Kassin, 2012) disregard the influence that false confessions exert and still render guilty verdicts. Thus, the overwhelming majority of suspects who falsely confess and go to trial are convicted (Drizin & Leo, 2004). One possible explanation for how this phenomenon violates the commonsense belief that people do not behave in ways counter to their self-interest stems from the fundamental attribution error.

#### Fundamental Attribution Theory

The fundamental attribution error suggests that people typically underestimate situational influences when making causal attributions for other people’s behavior (Gilbert & Malone, 1995; Ross, 1977, 2018). Regardless of whether this lack of situational attributions is made out of efficiency to reserve cognitive resources (Gilbert et al., 1988; Kubota et al., 2014) or because of people’s lack of awareness of the situational influences that impact others’ behavior, people have a tendency to attribute others’ behavior to internal causes. Jurors are no different.

The process of arriving at a situational explanation for people’s behavior is cognitively demanding, and appreciating that a confession resulted from situational pressures is especially difficult for jurors. Consider the following sequence with the understanding that, if at any point the sequence is disrupted, a person is less likely to make a situational attribution. First, jurors need to observe the situation (“*Suspect is being interrogated*”; Gilbert & Malone, 1995). Second, they need to be aware of the situational factors that a suspect is experiencing—an ambitious assumption given that jurors rarely observe the entirety of an interrogation (e.g., “*The questioning doesn’t seem to be too bad.*”). Third, jurors must consider their own behavioral expectations (e.g., “*No one would falsely confess so I expect the person won’t confess if they are innocent*”), which are often erroneous due to the accessible and available information that innocent people will never confess (Pyszczynski & Greenberg, 1987; Tversky & Kahneman, 1973). Finally, jurors must perceive and categorize the suspect’s behavior (e.g., “*The suspect is confessing*”).

Once a confession is perceived and categorized, jurors must make an attribution and apply dispositional (e.g., “*Innocents do not confess, but he did, so he must be guilty*”) or situational (e.g., “*Innocents do not confess, but he did, so the interrogation must be coercive*”) reasons for the suspect’s behavior. Because each step in this process places cognitive demands on the observer—and because critical situational information is often unavailable—jurors are more likely to default to dispositional explanations. As a result, they may conclude that the suspect is guilty rather than recognizing the role of situational pressures in producing the confession.

These attributional tendencies have important implications for how jurors evaluate confession evidence. Jurors may receive both dispositional information about a suspect (e.g., whether the suspect has an intellectual impairment) and situational information about the interrogation (e.g., whether the interrogation was objectively lengthy). Dispositional characteristics may be particularly salient because they provide an intuitive explanation for why a suspect falsely confessed (Henkel, 2008; Leo & Liu, 2009; Mindthoff et al., 2018). In contrast, appreciating the influence of situational characteristics is more difficult as it requires jurors to recognize how interrogation practices, such as inducing someone to believe they will experience a prolonged interrogation, can produce psychological pressure and increase the risk of false confession. In sum, jurors may place greater weight on dispositional information than on situational characteristics of the interrogation when evaluating the voluntariness of a confession and the defendant’s guilt (Leo & Liu, 2009; Scopelliti et al., 2018).

### 1.3. False Confessions and Perceptions of Exonerees

Sometimes innocent suspects falsely confess because of situational pressures that triers-of-fact struggle to appreciate, thereby facilitating their wrongful conviction. In some fortunate instances, however, innocent individuals who falsely confessed are exonerated of their wrongful conviction (Innocence Project, 2023; National Registry of Exonerations, 2026). Yet, being released and exonerated, although fortunate, often introduces these individuals into a world of persistent stigma (Clow & Leach, 2015; Norris et al., 2020; Westervelt & Cook, 2012). In fact, people desire more social distance from wrongly convicted exonerees and have negative emotions towards these exonerees compared to people in general (Clow & Leach, 2013)—effects that are even more pronounced toward exonerees who have falsely confessed (Clow & Leach, 2015; Kukucka & Evelo, 2019; Scherr et al., 2018).

In one study, exonerees who were convicted as the result of a false confession were more likely to be perceived as guilty, less competent, and less warm than exonerees wrongly convicted based on a mistaken eyewitness identification or jailhouse snitch (Clow & Leach, 2015). This may be the result of individuals believing that people who falsely confessed are more responsible for their conviction than individuals wrongfully convicted based on an eyewitness misidentification (Kukucka & Evelo, 2019; Kieckhaefer & Luna, 2022). Likewise, exonerees who falsely confessed are seen as less intelligent and more likely to suffer from mental health issues than exonerees convicted as the result of mistaken eyewitness identification (Scherr et al., 2018). The deep-rooted stigma individuals who falsely confess face is evident in various states laws that refuse to offer compensation to wrongfully convicted exonerees if the court believes that the exoneree assisted in their own conviction (e.g., offering a false confession; Compensation Statutes: A National Overview, 2024; Madrigal & Norris, 2022; Owens & Griffiths, 2012). Even post-exoneration, false confessions remain stigmatized and people default to attribute their past behaviors to dispositional causes rather than situational causes, leading to a delay in reintegration support (Scherr & Normile, 2022).

### 1.4. Fundamental Attribution Theory Revisited

Like triers-of-fact, laypeople fail to appreciate the situational factors that influence innocents to falsely confess (Henkel et al., 2008; Leo & Liu, 2009). As noted earlier, people are sometimes aware that false confessions occur, but they fail to recognize that innocents confess as the result of situational pressures present during an interrogation. This is in part because the general layperson does not believe that they would ever falsely confess to a crime regardless of the situation (Henkel et al., 2008). Therefore, although individuals self-report that extenuating circumstances could lead to someone falsely confessing, their judgments towards suspects may be tainted by their own disbelief that they would ever falsely confess (i.e., “*I would never falsely confess in this situation, so there is no way the suspect would either*.”). Instead, people may believe that the exoneree is at fault and responsible for their conviction.

## 2. Experiment 1

### 2.1. Overview

Prior research suggests that judges and jurors struggle to appreciate the situational influences that can shape innocents’ willingness to confess (Leo & Liu, 2009). Building on this work, the goal of Experiment 1 was to examine whether jurors fail to appreciate the coercive power of anticipated interrogation consequences in the context of false confessions. Although an individual may confess after experiencing a lengthy interrogation, an innocent suspect may also confess to avoid an anticipated negative consequence, such as the expectation of remaining in an interrogation for an extended period of time. Importantly, the fact that this pressure is anticipated rather than experienced may make it less apparent to observers, despite evidence that anticipated consequences can substantially influence decision-making and behavior (Madon et al., 2012, 2013). Thus, we examined whether jurors evaluate confessions differently when coercive pressure arises from an expectation of a prolonged interrogation versus the experience of a prolonged interrogation. If jurors do not adequately recognize these influences, they should be more likely to convict a suspect who has confessed.

Experiment 1 tested three conditional hypotheses and one exploratory hypothesis. First, the expectation hypothesis predicted that mock-jurors who read about a defendant who was actually interrogated for a long time (i.e., 6 h) would have lower likelihood of guilt judgments and would perceive the interrogation to be more coercive than mock-jurors who read about a defendant who was induced with the expectation that the questioning would last a long time. Second, the intelligence hypothesis predicted that participants who read about a defendant who was intellectually impaired would have a lower likelihood of guilt judgments and would perceive the interrogation to be more coercive and the confession less voluntary than participants who read about a defendant with average intelligence. The expectation-intelligence hypothesis predicted that the effects of the extended interrogation condition would be dependent on the intelligence of the defendant. Specifically, when defendants possessed lower intelligence, participants were expected to perceive the interrogation as relatively coercive regardless of whether the lengthy interrogation was anticipated or actually experienced because the defendant will be seen as especially vulnerable. In contrast, when defendants possessed average intelligence, we predicted that participants would rely more heavily on the interrogation circumstances, producing larger differences between the extended interrogation conditions. Lastly, an exploratory indirect analysis was conducted to examine the potential for participants’ perceptions of the interrogation to serve as an underlying mechanism that motivated the relationship between appreciation for situational influences and likelihood of guilt. Specifically, the juror situational appreciation test explored the potential for a moderated indirect relationship among length of interrogation as the predictor, exoneree intelligence as the moderator, appreciation for situational influences as the mediator, and participants’ likelihood of guilt as the outcome.

### 2.2. Method

#### 2.2.1. Participants

Initially, 276 participants were recruited from Amazon’s Mechanical Turk workforce (MTurk). To promote data quality, participation was restricted to MTurk workers with at least 5000 approved HITs and a minimal approval rating of 95%. Participants were further required to pass all attention and manipulation checks to be retained in the final sample. Overall, 23 participants were removed for failing attentional and manipulation check questions. Therefore, 253 MTurk participants were paid $1 to participate in the study. The number of participants was chosen based on a power analysis detecting small-to-medium-sized effects at α < 0.05 with 80% power (Cohen’s *f* = 0.18). Participants ranged from young adults to older adults (Ages: 21–74, *M_age_* = 39.72) and were approximately evenly split between women (54%) and men (46%). Participants were mostly White (75%), Black (11%) and Asian or Pacific Islander (10%). All participants were US citizens and Native-English speakers.

#### 2.2.2. Design

The study was a 2 (extended interrogation: expected length vs. actual length) x 2 (intelligence: average vs. intellectually impaired) between-subjects design. Participants were randomly assigned to read one of four modified transcripts from Kassin and Neumann (1997) detailing the trial of Charlie, a man accused of murdering his wife and neighbor. Participants in the expected length conditions read that Charlie, during a 1.5 h long interrogation, believed he would not be able to leave the interrogation unless he confessed, whereas participants in the actual length conditions read that Charlie was interrogated for 6 h, but no expectations were mentioned. In addition, participants in the average intelligence conditions were informed that Charlie had an IQ of 100, whereas participants in the intellectually impaired conditions read that Charlie had an IQ of 70. Participants provided their likelihood of guilt judgments for Charlie and their perceptions of the interrogation. Finally, participants responded to informational and attentional check items, manipulation check items, and provided demographic information.

#### 2.2.3. Transparency and Openness

We report how we determined our sample size, all data exclusions, all manipulations, and all measures in the study. All data and syntax are available at https://osf.io/aeqpd/overview?view_only=7f0e51df487c4f9db44f0ec58a674f85 (accessed on 30 June 2026). Data were analyzed using SPSS Version 28 (SPSS: An IBM Company, Armonk, NY, USA) and figures were created using Excel 2020 (Microsoft Corporation, Redmond, WA, USA). The study’s design and its analyses were not pre-registered.

#### 2.2.4. Measures

##### Trial Transcripts

The case summary was approximately 12 pages in length and written in the style of trial transcript. Each participant was randomly assigned to read one of four summaries involving Charlie Wilson, a man accused of murdering his wife and neighbor. Extended interrogation (expected vs. actual) and Charlie’s intelligence (average vs. impaired) were manipulated in the trial summaries. However, the material presented to participants included the same background information across all conditions, including the crime Charlie was accused of committing and the testimonies from both the prosecution and the defense.

##### Likelihood of Guilt Judgments

Participants rated how likely Charlie was guilty on a 0–100 scale (0 = *Charlie not guilty at all*, 100 = *Charlie Definitely guilty*).

##### Perceptions of the Interrogation

Participants were asked a variety of questions relating to their perceptions of the interrogation. Participants rated on a scale of 1 (*not very coercive*) to 9 (*very coercive*) how (a) coercive the interrogation was, (b) how aggressive the interrogator was, (c) how hostile the interrogation was, (d) how voluntary the confession was, and (e) how appropriate the entire interrogation was. Items d and e were reverse coded. These items were selected to assess participants’ overall perceptions of interrogation coerciveness, with each item representing a related, but unique, indicator of that broader evaluation. All items were summed together with higher scores indicating that participants perceived the interrogation to be more coercive. The composite demonstrated good reliability (*α* = 0.89).

##### Appreciation for Situational Influences

Participants provided answers to questions that gauged their appreciation of situational and dispositional factors. These questions were adapted from previous research (Morgan et al., 2010; α = 0.73). Participants were asked to rate on a scale of 1 (*not at all*) to 7 (*completely*) (a) to what extent was the confession under Charlie’s control, (b) to what extent Charlie could have acted another way during the interrogation, (c) to what extent was Charlie’s confession an inevitable and uncontrollable consequence of the interrogation, and (d) to what extent was Charlie’s behavior during the interrogation due to aspects of the situation he could not personally control. The first two items (i.e., a and b) were reverse coded. All items were then summed together with higher scores indicating stronger situational attributions (i.e., more appreciation for situational influences). The composite demonstrated acceptable reliability (*α* = 0.75).

##### Information and Attention Checks

A series of checks were used to assess participants’ recollection of critical information conveyed in the trial summaries using two multiple choice questions. The first question assessed participants’ ability to recall Charlie’s intelligence level. The second question assessed whether participants recalled the actual duration of the interrogation (i.e., whether he was interrogated for 6 h or 1.5 h). There were also four attention check questions placed throughout the survey that required participants to select a specific response to make sure participants were not mindlessly answering questions. For example, participants may have had to select “*Option A*” from a list of four options.

##### Demographics

A series of demographic questions was used to collect information about participants’ age, sex, race, and residence.

#### 2.2.5. Procedure

Participants were recruited using Amazon’s MTurk website. Interested participants were redirected to SurveyMonkey where they were presented with an online consent form. Informed consent was obtained from all participants involved in the study. Upon consent, participants were randomly assigned to read one of four trial summaries. After reading the trial summaries, participants provided their likelihood of guilt judgments, appreciation for situational influences, and perceptions of the interrogation. Finally, participants responded to informational and attentional check items as well as provided demographic information. It is important to note that participants were *not* able to go back and reread the transcript while answering these questions. They were then debriefed and paid for their participation.

### 2.3. Results

#### 2.3.1. Preliminary Analyses

##### Attention and Manipulation Checks

Participants were informed in the advertisement of the study and in the informed consent form that they would only be paid if they correctly answered the manipulation and attention check questions. This was in line with Amazon’s policy of only paying participants who provide high integrity data. As a result, data were only collected from participants who correctly answered the manipulation and attentional check questions. Of the initial 276 participants recruited, 23 were removed for providing low integrity data. Therefore, the rest of the analyses are based on the responses of the remaining 253 participants.

##### Skewness of Scales

Three tests for skewness were conducted in order to determine if the scale variables (i.e., likelihood of guilt, judgments of coercion, and appreciation for situational influences) were ordinal approximations of continuous variables. Because of the robustness of ANOVA tests, variables were considered substantially skewed if the ratio of skewness to its standard error was greater than the absolute value of 3. Results indicated that neither judgments of coercion (Skewness/*SE* = −2.60) nor appreciation for situational influences (Skewness/*SE* = −1.13) were substantially skewed. However, likelihood of guilt (Skewness/*SE* = −6.86) was substantially, negatively skewed. As a result, the data for likelihood of guilt were transformed using a logarithmic transformation after adding a constant of 1 to every score in order to remove all values of zero. Nevertheless, all findings using the transformed variable were the same as the findings for the original variable. Therefore, all descriptive statistics (e.g., means, standard deviations, coefficients, etc.) and inferential statistics for the likelihood of guilt variable are shown in their original, raw form for ease of interpretation.

#### 2.3.2. Main Analyses

##### Likelihood of Guilt Judgments

**Figure 1:**
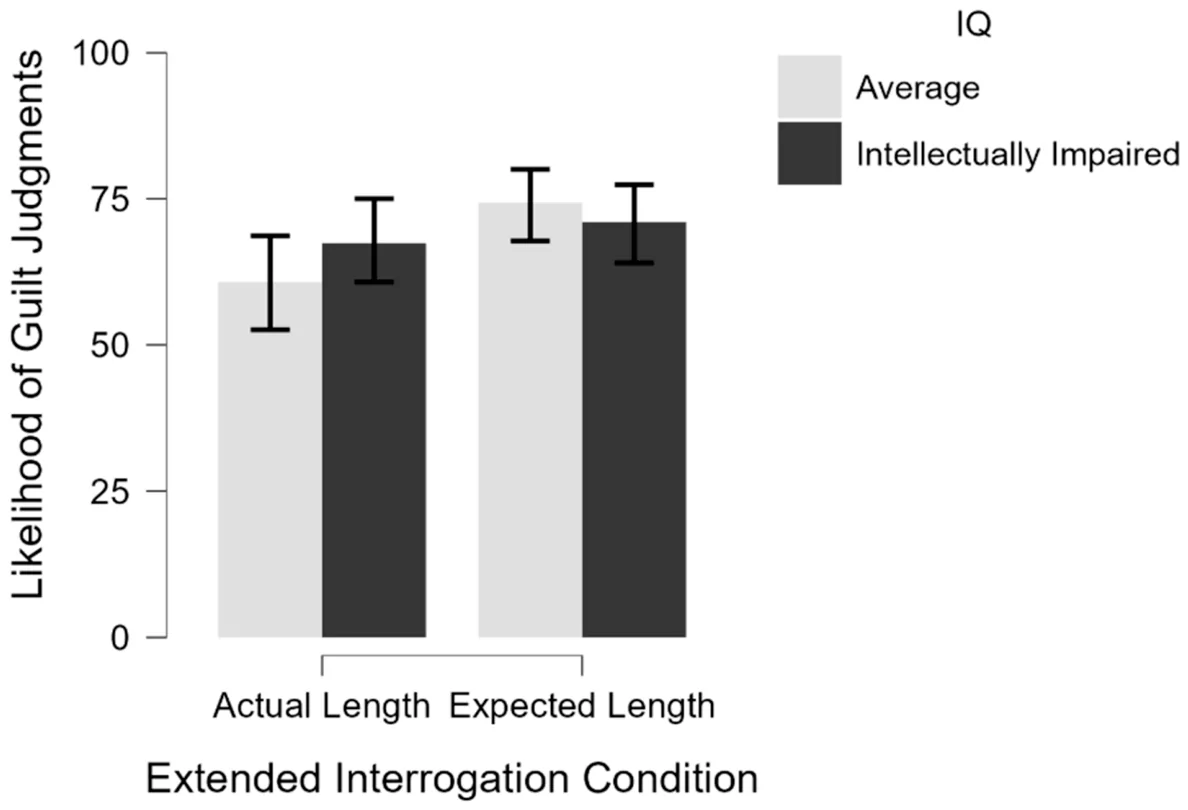
Mean likelihood of guilt ratings towards defendant in Experiment 1. Note. Error bars represent 95% confidence intervals.

To test participants’ judgments of guilt, we conducted a between-subjects factorial Analysis of Variance (ANOVA) with the continuous variable likelihood of guilt judgments as the dependent variable. Results revealed a significant main effect of extended interrogation, *F*(1, 249) = 5.78, *p* = 0.017, Cohen’s *d* = 0.29, 95% CI *d* [0.05, 0.55]. More specifically, participants who read about an expected length interrogation viewed the participant as more likely to be guilty (*M* = 72.61, *SD* = 27.38) than participants who read that the interrogation actually lasted six hours (*M* = 64.19, *SD* = 29.17; see Figure 1). However, there was no main effect of intelligence as there was no difference in guilt judgments between mock jurors who read that the defendant was intellectually impaired (*M* = 69.42, *SD* = 27.81) and those who read that the defendant had an average IQ (*M* = 67.90, *SD* = 29.29), *F*(1, 249) = 0.23, *p* = 0.634, Cohen’s *d* = 0.05, 95% CI *d* [−0.19, 0.30]. Furthermore, there was no extended interrogation × intelligence interaction effect, *F*(1, 249) = 1.91, *p* = 0.168, *η_p_*^2^ = 0.008, 90% CI for *η_p_*^2^ [0.000, 0.029].

##### Judgments of Coercion

**Figure 2:**
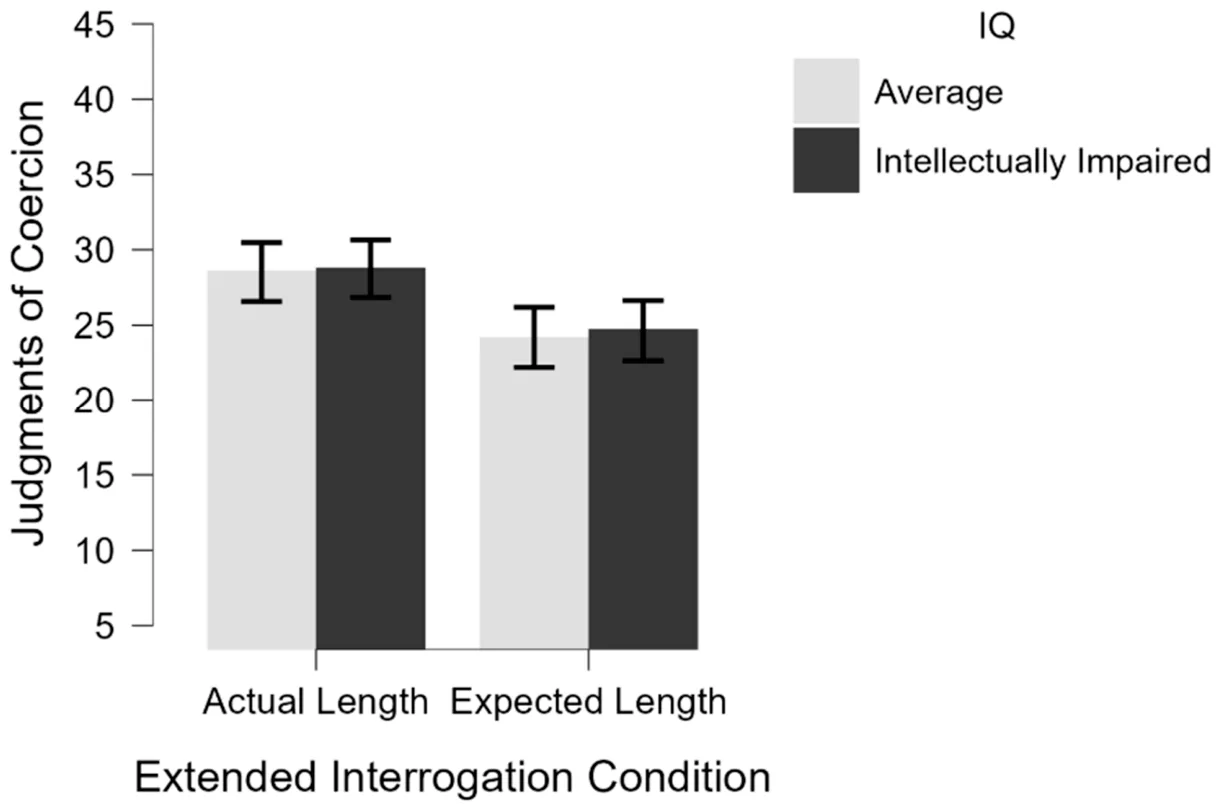
Mean ratings for judgments of coercion during interrogation in Experiment 1. Note. Possible Range: 5–45. Higher values indicate that participants believed the interrogation of Charlie was more coercive. Error bars represent 95% confidence intervals.

In order to examine the influences of the extended interrogation factor and intelligence of the defendant on mock-jurors’ perceptions of the interrogation, we conducted a between-subjects factorial ANOVA with the participants’ summed composite of their judgments of coercion as the dependent variable. There was a significant main effect of extended interrogation. Participants in the expectation group viewed the interrogation as less coercive (*M* = 24.48, *SD* = 8.18) than participants in the actual group (*M* = 28.73, *SD* = 7.87), *F*(1, 248) = 17.45, *p* < 0.001, Cohen’s *d* = −0.53, 95% CI *d* [−0.78, −0.28] (see Figure 2). However, there was no significant difference between participants who read that the defendant was intellectually impaired (*M* = 26.59, *SD* = 8.47) and those who read that the defendant was of average intelligence (*M* = 26.30, *SD* = 8.14; see Figure 2), *F*(1, 248) = 0.13, *p* = 0.719, Cohen’s *d* = 0.03, 95% CI *d* [−0.21, 0.28]. Moreover, there was no extended interrogation × intelligence interaction effect, *F*(1, 248) = 0.04, *p* = 0.851, *η_p_*^2^ < 0.001, 90% CI for *η_p_*^2^ [0.000, 0.016].

##### Exploratory Indirect Model

**Figure 3:**
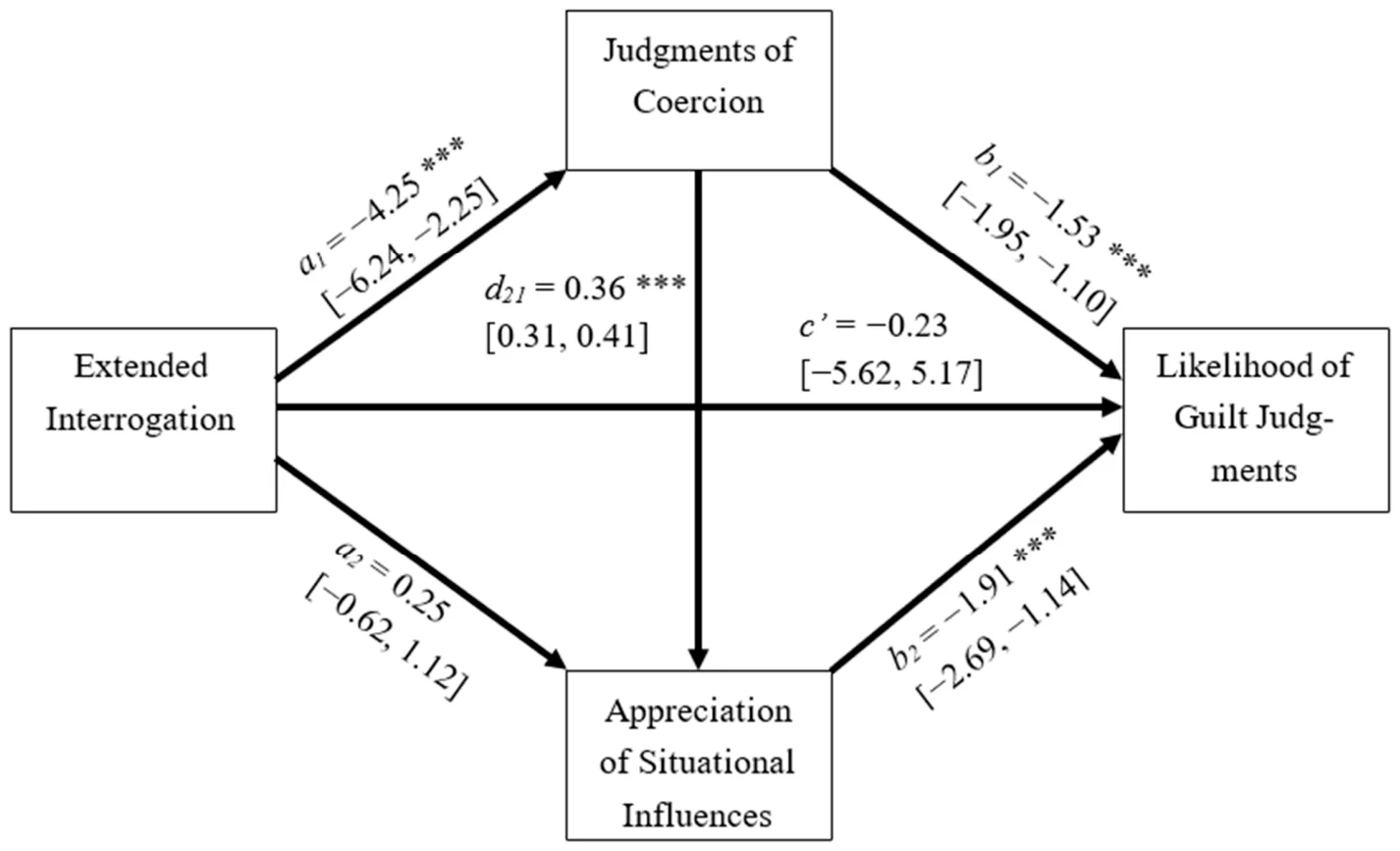
Serial mediation model testing the indirect effects of extended interrogation on likelihood of guilt judgments. Note. Confidence intervals based on 5000 bootstrapped samples. Extended Interrogation (0 = Actual Length, 1 = Expected Length). *** *p* < 0.001.

A moderated-mediation model with extended interrogation as the predictor, intelligence as the moderator, appreciation for situational influences as the mediator, and likelihood of guilt judgments as the outcome was originally planned. However, because we did not find a significant extended interrogation x intelligence interaction, we opted to explore an indirect model that was supported by our initial ANOVA results. Hence, we conducted a serial mediation analysis with extended interrogation as the predictor, judgments of coercion and appreciation of situational influences as serial mediators, and likelihood of guilt as the outcome (see Figure 3). Judgments of coercion were predicted to come before appreciation of situational influences based on the fundamental attribution theory. Namely, we reasoned that participants who found the interrogation to be more coercive would be more likely to appreciate the situational influence of said interrogation.

The analysis was conducted using PROCESS Model 6 (Hayes, 2018). Unstandardized direct effects were computed for each of 5000 bootstrapped samples. Mirroring the observed effects of the ANOVA presented earlier, results indicated a significant, negative relationship between extended interrogation and judgments of coercion, *b* = −4.25, *p* < 0.001, *R*^2^ = 0.07, 95% CI [−6.24, −2.24]—when participants read that the defendant confessed because of an expectation, they found the interrogation to be less coercive than participants who read that the defendant spent an extended period of time in the interrogation. Moreover, there was a significant, positive relationship between judgments of coercion and appreciation of situational influences, *b* = 0.36, *p* < 0.001, *R*^2^ = 0.44, 95% CI [0.31, 0.42]. Specifically, participants who found the interrogation to be more coercive were more likely to appreciate the situational factors that led to the defendants’ confession. Furthermore, there was a significant, negative relationship between appreciation of situational influences and likelihood of guilt in that participants who had more appreciation for situational factors found the defendant less likely to be guilty, *b* = −1.91, *p* < 0.001, *R*^2^ = 0.46, 95% CI [−2.69, −1.14].

Bootstrapped analyses indicated that the indirect relationship between extended interrogation and likelihood of guilt was serially mediated by judgments of coercion and appreciation of situational influences, *b* = 2.94, 95% CI [1.33, 4.87]. That is, participants viewed the interrogation of defendants who confessed as the result of an expectation to be less coercive than the interrogation of defendants who actually experienced a lengthy interrogation. This decrease in judgments of coercion led to less appreciation for situational influences which, consequently, led to higher likelihood of guilt judgments.

For readers curious about the model in which participants’ appreciation for situational influences predicts judgments of coercion, we reran the serial mediation analysis with appreciation for situational influences preceding judgments of coercion. Results revealed the same effects and patterns mentioned above.

### 2.4. Discussion

Experiment 1 evaluated whether mock-jurors could appreciate the coerciveness and power of expectations on suspects’ confession decision-making during an interrogation. The results of Experiment 1 demonstrated that mock-jurors found defendants induced with an expectation that the interrogation would last indefinitely to be more guilty than defendants who were not induced with an expectation (but were actually interrogated for a lengthy period of time). Furthermore, interrogations that included the induction of expectations were seen as less coercive than the longer interrogations. Hence, mock-jurors deemed suspects induced with an expectancy as more culpable and the interrogation as less coercive, suggesting that participants did not appreciate the social influences of the interrogation and, consequently, were highly confident in suspects’ guilt. The results from Experiment 1 provide evidence that appreciation for situational influences is related to likelihood of guilt judgments: lower degrees of appreciation for situational influences were associated with weaker judgments of interrogation coercion, resulting in higher likelihood of guilt ratings. Interestingly and unexpectedly, the intelligence of the defendant, a robust risk-factor associated with false confessions, had no effect on mock-jurors’ perceptions of guilt or coerciveness of the interrogation.

Experiment 1 extends our understanding of how people perceive false confessions. Mock-jurors, while appreciating the effect that length of an interrogation has on false confession rates, failed to appreciate that expectations can also lead to false confessions. Thus, innocent defendants who falsely confessed because of an expectation may be at additional risk for wrongful conviction compared to those defendants who falsely confessed where no expectation was present but the questioning lasted objectively longer. Critically, in both situations, the defendant was innocent.

Although wrongful convictions do occur, in some fortunate instances the wrongly convicted individual is eventually exonerated. These exonerees must then assimilate back into society. Although exonerees are stigmatized, especially those who falsely confess, we do not know whether certain factors associated with the false confession can result in particularly high degrees of stigma. Experiment 2 examined the idea of whether laypeople appreciate the influence of expectations on those who have been exonerated of a wrongful conviction stemming from a false confession.

## 3. Experiment 2

Generally, exonerees face stigma upon attempting to reintegrate (e.g., Faison & Smalarz, 2020). For instance, people are reluctant to fully support reintegration services for wrongfully convicted exonerees (Clow & Leach, 2015), especially when the exoneree falsely confessed (Kukucka & Evelo, 2019; Scherr et al., 2018). Those wrongly convicted and exonerated struggle to find housing (e.g., Zanella et al., 2020), experience unique mental health issues (e.g., Kukucka et al., 2022), and are discriminated against when attempting to find jobs (e.g., Kukucka et al., 2020). Similarly to how attributions of false confession evidence in the courtroom increase the likelihood of a wrongful conviction, post-exoneration, people may believe that exonerees who falsely confessed are responsible for their conviction and, therefore, undeserving of support.

Although members of the public may not always be aware of the specific circumstances surrounding an interrogation when learning about an exoneree, information about interrogation experiences may be available through news reports, trial testimony, interrogation recordings, and compensation lawsuits. Thus, examining how people interpret information about interrogation circumstances provides insight into how situational factors surrounding a confession can influence perceptions of an exoneree’s culpability and deservedness of aid. In Experiment 2, we examined whether interrogation expectancy and defendant intelligence influenced participants’ appreciation of situational influences during the interrogation and their subsequent judgments of exoneree guilt and support for reintegration.

Although many of the procedures mirrored those of Experiment 1, Experiment 2 examined a subsequent step by addressing peoples’ willingness to support wrongly convicted exonerees. In addition, the results from Experiment 2 can help understand how the fundamental attribution error may lead people to maintain guilty beliefs towards wrongly convicted exonerees who falsely confessed despite the existence of exculpatory DNA evidence.

Experiment 2 tested three conditional hypotheses and one exploratory hypothesis. First, the actual length hypothesis predicted that participants in the actual condition would have lower guilt-judgment ratings and would be more likely to support reintegration services for wrongfully convicted exonerees than participants in the expected length condition. The intelligence hypothesis predicted that participants who read about an exoneree who was intellectually impaired would have lower guilt confidence ratings and would be more likely to support reintegration services than participants who read about an exoneree with average intelligence. The expected length intelligence hypothesis predicted that the effects of the length of the interrogation would be dependent on the intelligence of the exoneree, such that exonerees who were intellectually impaired and interrogated without being induced with an expectation of length would be accompanied by the lowest guilt ratings and subsequently receive the most support for reintegration services, while defendants with average intelligence who were induced with the expectation of a lengthy interrogation would be accompanied by the highest guilt ratings and lowest support for reintegration.

An exploratory moderated-mediation analysis was also conducted. The test examined the potential for participants’ appreciation for situational influences to serve as an underlying mechanism that motivated the relationship among expectancy presence, exoneree intelligence, and likelihood of guilt judgments. Specifically, the exoneree situational appreciation test explored the potential for a moderated-mediation indirect relationship among expectancy as the predictor, exoneree intelligence as the moderator, appreciation for situational influences as the mediator, and participants’ likelihood of guilt as the outcome.

### 3.1. Method

#### 3.1.1. Participants

Initially, 288 participants were recruited from Amazon’s Mechanical Turk workforce. Like Experiment 1, participation was restricted to MTurk workers with at least 5000 approved HITs and a minimal approval rating of 95%. Participants were further required to pass all attention and manipulation checks to be retained in the final sample. Overall, 27 participants failed to correctly answer attentional check questions (detailed below). Therefore, a final sample of 261 participants were recruited from Amazon’s Mechanical Turk workforce and paid $1.25 to participate in the study. The number of participants was chosen based on a power analysis detecting small-to-medium-sized effects (Cohen’s *f* = 0.18) at α < 0.05 with 80% power. Participants ranged from young adults to older adults (Ages: 21–70, *M_age_* = 35.63) and were mostly men (62%). In addition, participants consisted of mostly White (81%), Black (7%) and Asian or Pacific Islander (6%) individuals. All participants were US citizens and Native-English speakers.

#### 3.1.2. Design

The study was a 2 (extended interrogation: expected length vs. actual length) x 2 (intelligence: average vs. intellectually impaired) between-subjects design. Participants were randomly assigned to read one of four news stories regarding a wrongly convicted man named Charlie Wilson. The news story detailed that Charlie was wrongly convicted of second-degree murder after offering a false confession. While participants in the expected length conditions read that Charlie inferred that he will be held indefinitely unless he cooperated (and subsequently was interrogated for 1.5 h before confessing), participants in the actual length conditions read that Charlie was interrogated for 6 h. In addition, participants assigned to the average intelligence conditions were informed that Charlie had an IQ of 100 while participants assigned to the intellectually impaired conditions read that Charlie was intellectually impaired and had an IQ of 70. Regardless of condition, all participants read that Charlie was exonerated of his crime based on DNA testing. Participants answered questions regarding the likelihood of Charlie’s guilt and rated their willingness to support three reintegration services for Charlie (i.e., psychological counseling, job training, and career counseling). Participants’ perceptions of Charlie were also collected (e.g., how much participants pitied Charlie, how warm they believed Charlie to be, etc.). However, these variables were collected for a separate project; therefore, participants’ perceptions of Charlie’s character are not presented in the current manuscript. Finally, participants responded to attentional check items and completed the manipulation check items. Participants then provided demographic information and were thanked for their participation.

#### 3.1.3. Transparency and Openness

We report how we determined our sample size, all data exclusions, all manipulations, and all measures in the study. All data and syntax are available at https://osf.io/aeqpd/overview?view_only=7f0e51df487c4f9db44f0ec58a674f85 (accessed on 30 June 2026). Data were analyzed using SPSS Version 28 and figures were created using Excel 2020. The study’s design and its analyses were not pre-registered.

#### 3.1.4. Measures

##### News Story

Each participant was randomly assigned to read one of four fictional news stories about a wrongful conviction case involving Charlie. Extended interrogation (expected length vs. actual length) and Charlie’s intelligence (average vs. intellectually impaired) were manipulated in the fictional news story. However, the story included the same background information across all conditions by detailing that Charlie was wrongly convicted of second-degree murder. In addition, all participants read that Charlie was exonerated as the result of DNA evidence and the article stressed that Charlie was factually innocent of the crime.

##### Likelihood of Guilt Judgments

Participants rated how likely Charlie is guilty from 0 (*Charlie is not guilty at all*) to 100 (*Definitely Charlie guilty*).

##### Support for Reintegration Services

Like the approach used in previous research (Clow & Leach, 2013, 2015; Scherr et al., 2018), participants’ willingness to support reintegration efforts concerning *psychological counseling, career counseling,* and *job training* was assessed with three statements: *Charlie should get government-sponsored psychological counseling/career counseling/job training.* Participants rated how much they agreed with each statement from 1 (*Strongly disagree*) to 9 (*Strongly agree*). All items were summed together with higher scores indicating more support for reintegration services. The composite demonstrated excellent reliability (*α* = 0.91).

##### Appreciation for Situational Influences

Participants provided answers to questions that gauged their appreciation of situational and dispositional factors. Participants rated from 1 (*not at all*) to 7 (*completely*): (a) to what extent was the confession under Charlie’s control, (b) to what extent Charlie could have acted another way during the interrogation, (c) to what extent was Charlie’s confession an inevitable and uncontrollable consequence of the interrogation, and (d) to what extent was Charlie’s behavior during the interrogation due to aspects of the situation he could not personally control. The first two items were reverse coded. All items were summed together with higher scores indicating stronger situational attributions (i.e., more appreciation for situational influences). The composite demonstrated good reliability (*α* = 0.82).

##### Information and Attention Checks

We used two multiple choice questions to assess participants’ recollection of critical information conveyed in the news stories. The first question assessed participants’ ability to recall Charlie’s intelligence level. The second question assessed whether participants recalled the actual duration of Charlie’s interrogation (i.e., whether he was interrogated for 6 h or 1.5 h). There were also four attentional check questions placed throughout the survey that required participants to select a specific response to make sure participants were not mindlessly answering questions. For example, participants may have had to select “*Option A*” from a list of four options.

##### Demographics

A series of demographic questions was used to collect information about participants’ age, sex, race, and country of residence.

#### 3.1.5. Procedure

Participants were recruited using Amazon’s MTurk website. Interested participants were redirected to Qualtrics where they were presented with an online consent form. Informed consent was obtained from all participants involved in the study. Upon consent, participants were randomly assigned to read one of the four news stories. After reading the news story, participants provided their perceptions of Charlie’s character and the likelihood that Charlie was guilty. Next, participants responded to manipulation-check, character-judgment, and appreciation-for-situational-influences questions. It is important to note that participants were unable to go back and read the news story at this point to ensure that participants could answer the questions based on their own memory of the news story. Then, participants indicated their willingness to support three reintegration services: (a) psychological counseling, (b) career counseling, and (c) job training. Finally, participants were presented with demographic questions and then debriefed and paid for their participation.

### 3.2. Results

#### 3.2.1. Preliminary Analyses

##### Attention and Manipulation Checks

Participants were informed in the advertisement of the study and in the informed consent form that they would only be paid if they correctly answered the manipulation and attention check questions. This was in line with Amazon’s policy of only paying participants who provide high integrity data. As a result, data were only collected from participants who correctly answered the attentional and manipulation check questions.

##### Skewness and Data Transformation

Three skew tests were conducted in order to determine if the scale variables (i.e., likelihood of guilt, reintegration support, and appreciation for situational influences) were ordinal approximations of continuous variables. Because of the robustness of ANOVA tests, variables were considered substantially skewed if the ratio of skewness to its standard error was greater than the absolute value of 3. Results indicated that appreciation for situational influences was not substantially skewed (Skewness/*SE* = −1.66).

The variables likelihood of guilt (Skewness/*SE* = 14.66) and reintegration support (Skewness/*SE* = −9.30) were substantially skewed. As a result, the data for likelihood of guilt were transformed using a logarithmic transformation after adding a constant of 1 to every score in order to remove all values of zero. Nevertheless, all of the findings using the transformed variable were the same as the findings for the original variable. Therefore, all descriptive statistics (e.g., means, standard deviations, coefficients, etc.) and inferential statistics for the likelihood of guilt variable are shown in their original, raw form for ease of interpretation. Logarithmic transformations did not correct skewness for reintegration support, and therefore, non-parametric tests were conducted with this variable in order to verify parametric results.

#### 3.2.2. Main Analyses

##### Likelihood of Guilt

**Figure 4:**
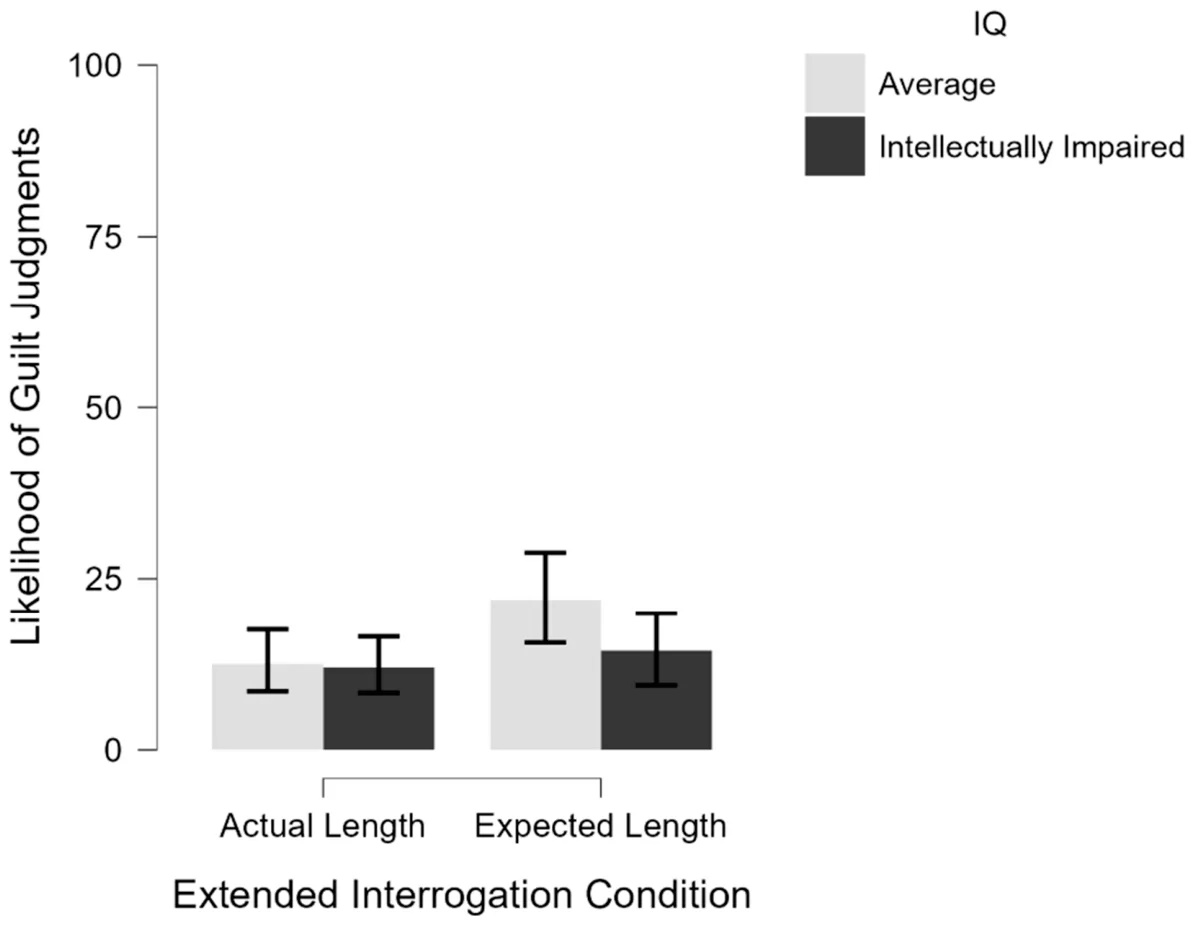
Mean likelihood of guilt ratings towards exonerees. Note. Higher values indicate higher perceptions of guilt. Error bars represent 95% confidence intervals.

A between-subjects factorial ANOVA was conducted in order to ascertain how expectations and exoneree intelligence influenced participants’ likelihood of guilt judgments. The actual length hypothesis predicted that participants in the actual condition would have lower guilt-judgment ratings and would be more likely to support reintegration services for wrongly convicted exonerees than participants in the expected length condition. However, the results did not support this prediction. There was no significant difference between participants in the actual (*M* = 10.37, *SD* = 15.19) and expected (*M* = 13.52, *SD* = 22.21) groups, *F*(1, 257) = 1.88, *p* = 0.172, Cohen’s *d =* 0.17, 95% CI of *d* [−0.08, 0.41] (see Figure 4). In addition, there was no support for the intelligence hypothesis—that is, there were no differences in the likelihood of guilt judgments from participants who read about an exoneree who was cognitively impaired (*M* = 12.07, *SD* = 19.56) and those who read that the exoneree was of average intelligence (*M* = 11.71, *SD* = 18.32), *F*(1, 257) = 0.02, *p* = 0.893, Cohen’s *d =* 0.02, 95% CI of *d* [−0.22, 0.26]. Moreover, results failed to support the *expected length intelligence hypothesis*—there was no significant interaction effect on guilt judgments, *F*(1, 257) = 0.88, *p* = 0.348, *η_p_*^2^ = 0.003, 90% CI for *η_p_*^2^ [0.000, 0.025].

##### Reintegration Support

**Figure 5:**
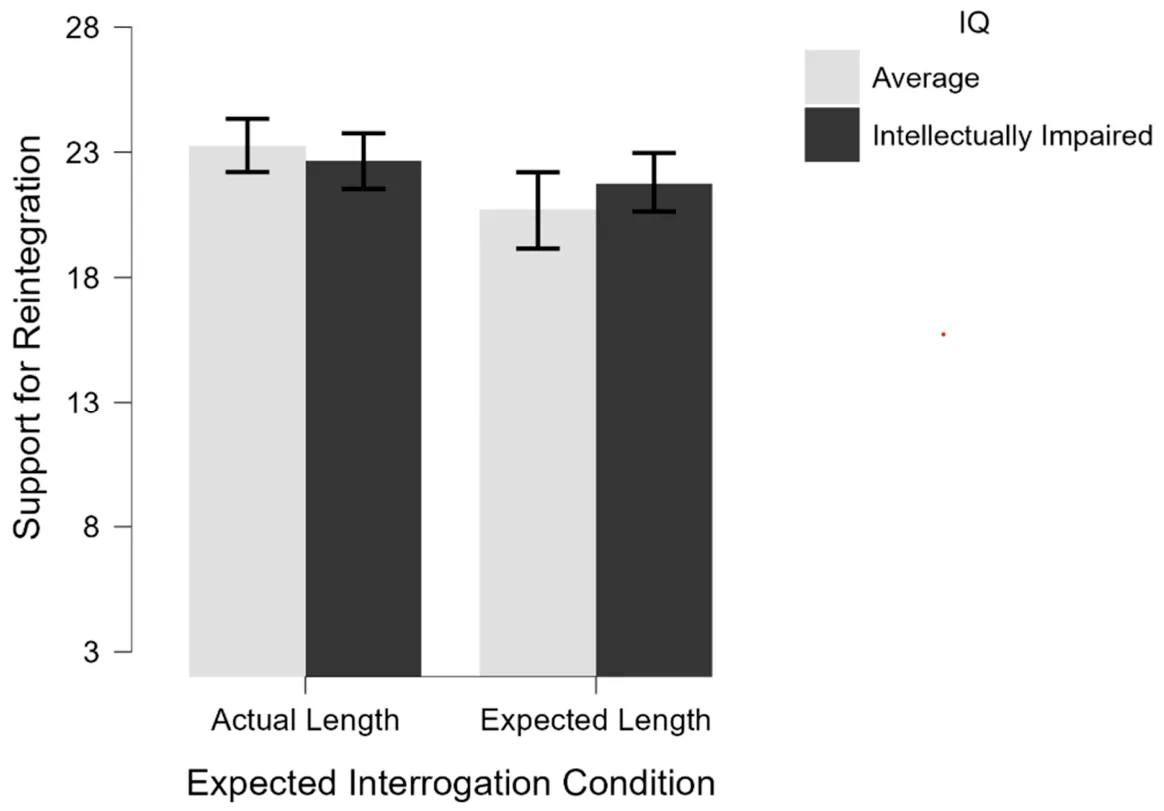
Mean support for reintegration services for exonerees. *Note.* Possible Range: 3–27. Higher values indicate more willingness to support services. Error bars represent 95% confidence intervals.

A between-subjects factorial ANOVA was conducted in order to ascertain how extended interrogation and exoneree intelligence affected participants’ support for reintegration services for the exoneree.^1^ Consistent with the actual length hypothesis, results revealed a significant main effect of extended interrogation, in that participants in the actual length group (*M* = 23.18, *SD* = 4.81) were significantly more likely to support reintegration services for the exoneree who falsely confessed after an actual lengthy interrogation compared to participants in the expected length group (*M* = 21.34, *SD* = 6.15), *F*(1, 257) = 7.43, *p* = 0.007, Cohen’s *d* = −0.33, 95% CI of *d* [−0.58, −0.09] (see Figure 5). However, there was again no support for the *intelligence hypothesis*—there was no difference in reintegration support for exonerees who were intellectually impaired (*M* = 22.36, *SD* = 5.06) and those with average intelligence (*M* = 22.22, *SD* = 6.07), *F*(1, 257) = 0.05, *p* = 0.828, Cohen’s *d =* 0.02, 95% CI of *d* [−0.22, 0.27]. There also was no support for the *expected length intelligence hypothesis* because no significant interaction effect was observed, *F*(1, 257) = 1.64, *p* = 0.201, *η_p_*^2^ = 0.006, 90% CI for *η_p_*^2^ [0.000, 0.032].

#### 3.2.3. Exploratory Appreciation Model

**Figure 6:**
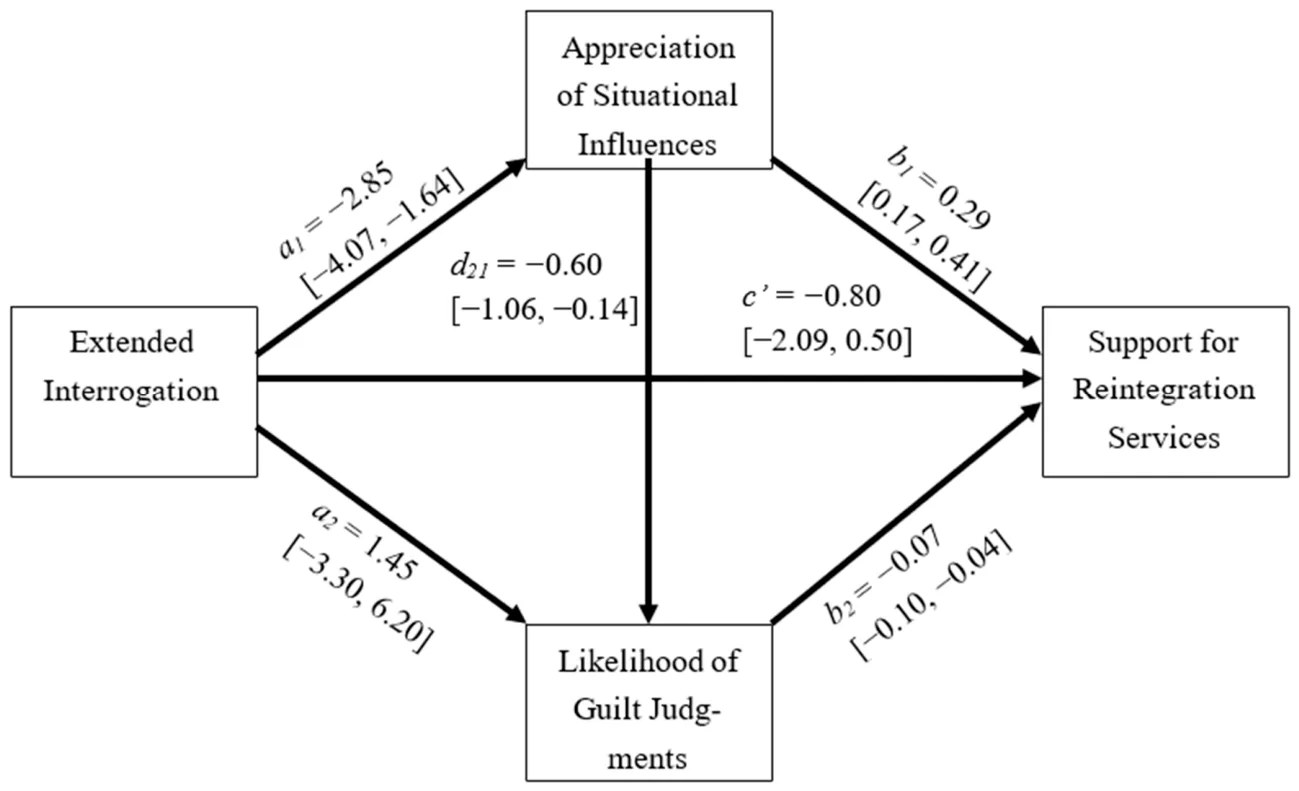
Serial mediation model testing the indirect effects of extended interrogation on support for reintegration services. *Note.* Confidence intervals based on 5000 bootstrapped samples. Extended Interrogation (0 = Actual Length, 1 = Expected Length).

A moderated-mediation model with extended interrogation as the predictor, intelligence as the moderator, appreciation for situational influences as the mediator, and likelihood of guilt judgments as the outcome was originally planned to be conducted using PROCESS Model 8. However, because there was no extended interrogation x intelligence interaction, we decided to not run the analysis as originally intended. Instead, we conducted a serial mediation analysis with extended interrogation as the predictor, appreciation of situational influences and likelihood of guilt as serial mediators, and reintegration support as the outcome (see Figure 6). Appreciation of situational influences was predicted to come before likelihood of guilt based on the fundamental attribution theory, as well as our findings from Experiment 1.

Unstandardized direct effects were computed for each of 5000 bootstrapped samples using PROCESS Model 6 (Hayes, 2018). Results indicated a significant, negative relationship between extended interrogation and appreciation for situational influences, *b* = −2.85, *p* < 0.001, *R*^2^ = 0.08, 95% CI [−4.07, −1.64]. Specifically, participants who read that the exoneree had confessed based on the expectation of a lengthy interrogation had less appreciation for situational influences than participants who read that the exoneree had confessed because they actually experienced a lengthy interrogation. Moreover, there was a significant, negative relationship between appreciation for situational influences and likelihood of guilt, *b* = −0.60, *p* = 0.011, *R*^2^ = 0.03, 95% CI [−1.06, −0.14]. That is, participants who had less appreciation for situational influences had higher likelihood of guilt judgments. Furthermore, there was a significant, negative relationship between likelihood of guilt judgments and support for reintegration services in that participants who had higher likelihood of guilt judgments offered less support for reintegration services, *b* = −0.07, *p* < 0.001, *R*^2^ = 0.17, 95% CI [−0.10, −0.04].

Bootstrapped analyses indicated that the relationship between extended interrogation and support for reintegration support was serially mediated by appreciation for situational influences and likelihood of guilt judgments, *b* = −0.12, 95% CI [−0.27, −0.03]. The relationship indicates that participants who read that the exoneree confessed based on an expectation had less appreciation for situational influences, and, in turn, a weaker appreciation for situational influences was associated with a higher likelihood of guilt judgments which, consequently, predicted less support for reintegration services such as psychological counseling and job training.

### 3.3. Discussion

In Experiment 2 we attempted to determine whether laypeople would fail to appreciate the power of expectations during an interrogation, and if this in turn would affect a person’s willingness to support reintegration services for a wrongly convicted exoneree. Initial results from Experiment 2 demonstrated no difference in the likelihood of guilt judgments regardless of whether expectations were induced during his interrogation or the exoneree’s intelligence. However, despite people generally perceiving the exoneree as not guilty (*M* = 11.93, *SD* = 18.97), exonerees who were induced with the expectation that the interrogation would last for a substantial period of time were judged less deserving of reintegration support than those who were not induced with an expectation (but were, in fact, questioned for a long period of time).

Exploratory indirect results suggested that people’s ability to appreciate situational influences is a meaningful motivator of their guilt judgments and willingness to support reintegration of exonerees who were wrongly convicted based on a false confession. Indeed, participants who read about the exoneree who was induced with expectations had less appreciation for situational influences, which in turn were associated with judgments that the exoneree was more likely to be guilty and, consequently, predicted less support for reintegration services. Although the resulting change in reintegration was small in magnitude, the findings are nevertheless important. Participants were explicitly informed that the exoneree was factually innocent, yet those in the expected length condition were less willing to support the exoneree’s reintegration into society. This finding is particularly concerning given the substantial challenges exonerees already face when attempting to reintegrate into society, including discrimination in employment (Kukucka et al., 2020), housing (Kukucka et al., 2021; Zanella et al., 2020), and healthcare (Kukucka et al., 2022), all of which could lead to exonerees committing a real crime and returning to prison (Shlosberg et al., 2014). Thus, even a modest reduction in support may compound the social and practical barriers that exonerees face as they rebuild their lives following a wrongful conviction.

## 4. General Discussion

Experiment 1 examined mock-jurors’ ability to appreciate the power of expectations on suspects’ confession decisions. Although the effects were modest in magnitude, mock-jurors were more likely to find the defendant who confessed after being induced with an expectation to be guiltier than a suspect not induced with an expectation, and the interrogation less coercive overall than one that included the inducement of an expectation. One possible reason mock-jurors arrived at these judgments is because they failed to appreciate the situational influences of the interrogation. That is, rather than attributing the confession to the coerciveness of the interrogation, mock-jurors appeared to attribute the confession to the defendant’s guilt.

Experiment 2 extended the results from Experiment 1 to judgments of those exonerated after a wrongful conviction. Specifically, Experiment 2 examined whether laypeople can appreciate the power of expectations when evaluating exonerees (who were objectively exonerated by DNA evidence) and their willingness to support reintegration services. Contrary to hypotheses, participants did not differ in their persevering guilt judgments of exonerees who falsely confessed after being led to expect a lengthy interrogation and exonerees who falsely confessed following an actual 6 h interrogation. However, when appreciation for situational influences was introduced into the analyses, expectations indirectly influenced persevering guilt judgments. That is, participants who read about an exoneree who falsely confessed after being led to expect a lengthy interrogation demonstrated less appreciation for situational influences and, in turn, perceived the exoneree as more likely to be guilty than participants who read about an exoneree who experienced an actual, lengthy 6 h interrogation. Moreover, participants who read about an exoneree who confessed after being led to expect a lengthy interrogation were less willing to support reintegration services than participants who read about an exoneree who experienced a 6 h interrogation.

### 4.1. Theoretical Implications

The findings for Experiment 1 revealed that mock-jurors are unable to appreciate the situational influence that expectations may have on confession decision-making. Individuals fail to make situational attributions because it expends valuable cognitive resources (Gilbert et al., 1988; Kubota et al., 2014). Indeed, in order for jurors to make a situational attribution they must first: (1) observe a situation, (2) be aware that a situational factor is occurring, and then (3) consider their own behavioral expectations, before (4) categorizing the suspect’s behavior (Gilbert & Malone, 1995). Results from Experiment 1 highlight how this chain of events breaks down, thereby decreasing the likelihood that jurors will apply a situational attribution for defendants’ behavior. The observed effects suggest that, although triers-of-fact can appreciate how a lengthy interrogation could lead to a false confession, the power of expectations does not meet the threshold that needs to be reached in order for jurors to attribute a false confession to a situational reason.

Like Experiment 1, the results from Experiment 2 indicated a general difficulty in appreciating situational influences such as expectations on confession decision-making. Expectancies did not directly influence individuals’ likelihood of guilt judgments when people were led to believe that an exoneree falsely confessed after being induced with an expectation compared to when no expectation was present. Although previous research has shown that individuals view exonerees who falsely confess as more likely to be guilty compared to exonerees convicted based on mistaken eyewitness identifications or a jailhouse snitch (Clow & Leach, 2015), the results from Experiment 2 suggest that the reason for a false confession may be overshadowed by the presence of a false confession.

Deservedness of reintegration support, however, was influenced by whether the confession was expectancy-induced in that those induced with expectations received less support than those who were not induced with expectations but experienced a long interrogation. One possible reason for this could be that the effect of long interrogations on confessions may be easier to appreciate and laypeople blame the criminal justice system for misconduct leading to instances of wrongly convicting an innocent suspect (e.g., Kukucka & Evelo, 2019). For example, it may be the case that the individuals who read about a lengthy interrogation that led to a false confession may find the criminal justice system more at fault and, therefore, should provide reintegration for the exoneree who was falsely imprisoned. On the other hand, they place more blame on the exoneree and attribute less to misconduct when false confessions are expectancy-induced.

### 4.2. Policy Implications

The results from Experiment 1 highlight the importance of allowing expert witnesses to testify about the influence of situational factors present during interrogations—an idea consistent with research establishing that jurors have a difficult time understanding that false confessions occur (Henkel et al., 2008). Allowing experts to testify would help jurors understand how situational factors influence someone to falsely confess. Along with consistent calls to mandate the recording of all interrogation interactions, we further suggest that an expert should be present during the viewing of a video-taped interrogation so that the expert could elaborate on what is occurring and, in the case of expectations, explain how the tactic can induce innocents into giving a false confession. This is especially true in cases that involve vulnerable populations, including those who are cognitively immature and thus may be more likely to act in an impulsive manner (Owen-Kostelnik et al., 2006).

The findings of Experiment 2 further establish the difficulties that some innocents face upon being exonerated and assimilating into society. Only 38 states—as well as Washington D.C. and the US federal government—have any statutes that address restitutions for wrongly convicted exonerees (Compensation Statutes: A National Overview, 2024). Unfortunately, some of these states have caveats that prevent exonerees from collecting restitutions if they contributed to their own conviction (i.e., falsely confessed or falsely pled guilty). Furthermore, some states require exonerees to go to trial in order to argue for restitutions, and so decisions regarding services and financial compensation are made on a case-by-case basis. The results from Experiment 2 support the notion that policies should guarantee all exonerees access to reintegration services. This would prevent biases from allowing some exonerees access to services while denying access to others. Guaranteeing access to aids will allow even those who falsely confessed because they expected a lengthy interrogation to receive the same access to services as those who falsely confessed as the result of an actual lengthy interrogation and all other individuals who have been exonerated of a wrongful conviction.

### 4.3. Limitations and Future Directions

Although several promising effects were observed, there are important limitations that future research should further examine. For example, it was surprising that the intelligence of the defendant in Experiment 1 did not influence guilt or interrogation coercion judgments. Indeed, previous research has shown that laypeople are generally aware that dispositional factors, including the intelligence of a suspect, have a moderate-to-large effect on false confession rates (Henkel et al., 2008). Perhaps it was the case that even though the mock-jurors knew there was a difference in intelligence, they generally believed both defendants had, overall, less-than-average intelligence. Research has shown that people believe criminals to be uneducated and unintelligent (Reed & Reed, 1973). This is especially true for criminals accused of committing a violent crime (O’Connor, 1984). In addition, individuals who falsely confessed to a crime are seen as less intelligent and therefore more likely to be guilty than those who did not falsely confess (Scherr et al.,2018). Future research would benefit from not only asking mock-jurors to offer intelligence perceptions, but to give explanations for these perceptions to better understand this phenomenon.

The lack of a difference in guilt judgments towards exonerees who were induced with the expectation of a lengthy interrogation and exonerees who were actually interrogated for a long time may be the result of a floor effect in Experiment 2. Indeed, about 73% of participants (*n* = 191) had guilt judgments less than or equal to 10. The use of DNA evidence as the exonerating piece of evidence may be the cause of this floor effect. Because of the strong impact of DNA evidence as an exonerating piece of evidence, it would behoove researchers to examine perceptions of exonerees without explicitly stating the evidence that led to exoneration. That is to say, future studies could keep the reason for exoneration ambiguous or have one group mention DNA and one group ambiguous to further examine the role that the exonerating factor has on laypeople’s judgments of exonerees’ guilt.

Although the diverse sample characteristics in Experiment 1 is similar to the demographic breakdowns of jury-eligible citizens in the USA, participants in Experiment 2 were noticeably more male-identifying and White than the overall US population. Therefore, we urge caution when considering the generalizability of the results to more diverse populations across a variety of social and political identities. A promising direction for future research would be to explore how demographics of triers-of-fact may influence their appreciation for situational influence that could lead to a false confession during an interrogation. Indeed, an intersectional approach investigating the interactions between race, gender, class, sexuality, and other social identities could allow for a better understanding of people’s perceptions of the wrongfully convicted and their subsequent support for reintegration services.

Finally, although our manipulation checks assessed participants’ memory for the actual duration of the interrogation, they did not directly assess their memory for the anticipated duration. Thus, although participants in the expected interrogation condition were provided with information indicating that the suspect believed the interrogation would continue indefinitely unless they confessed, we cannot be certain whether all participants retained this information when making subsequent judgments. Future research should assess memory for both actual and anticipated interrogation duration to more directly establish that participants retained the expectancy manipulation.

## 5. Conclusions

Suspects brought into an interrogation are often presented with expectancy-related tactics that meaningfully influence their decision-making and behavior. Interrogators maintain a guilt presumption towards suspects and primarily focus on extracting confessions that can lead innocents to falsely confess to a crime to escape an aversive environment. This is especially seen in instances in which interrogators induce expectancies and imply to suspects that they cannot leave unless he or she “gives the police what they want.” A confession from an innocent in any of these situations, even if it is later recanted, is damning evidence because triers-of-fact struggle to appreciate the power of expectations. Even in the fortunate instances of a wrongfully convicted person being exonerated, the stigma persists against someone who falsely confessed simply because of an expectation. The significant observed effects add to a growing literature that underscores the importance of bringing about awareness regarding false confessions, their influence on trial verdicts, and their lasting effects even upon exoneration (Scherr et al., 2020).

## Data Availability

All data and syntax can be found on the Open Science Framework website at https://osf.io/aeqpd/overview?view_only=7f0e51df487c4f9db44f0ec58a674f85 (accessed on 30 June 2026).

## Note

1 Because the data for reintegration support was negatively skewed we ran two Mann–Whitney U tests. The results from these non-parametric tests mirror the reported ANOVA results.
